# Reconstructing Kaolinite Compounds for Remarkably Enhanced Adsorption of Congo Red

**DOI:** 10.3390/molecules29092121

**Published:** 2024-05-03

**Authors:** Ting Liu, Xinle Li, Hao Wang, Mingyang Li, Hua Yang, Yunhui Liao, Wufei Tang, Yong Li, Fang Liu

**Affiliations:** 1 Hunan Engineering Technology Research Center for Comprehensive Development and Utilization of Biomass Resources, College of Chemistry and Bioengineering, Hunan University of Science and Engineering, Yongzhou 425199, China; liuting@huse.edu.cn (T.L.); lixinle@huse.edu.cn (X.L.); zengy@huse.edu.cn (H.W.); limingyang@huse.edu.cn (M.L.); yangh@huse.edu.cn (H.Y.); liaoyunhui@huse.edu.cn (Y.L.); 2 CAS Key Laboratory of Mineralogy and Metallogeny, Guangdong Provincial Key Laboratory of Mineral Physics and Materials, Guangzhou Institute of Geochemistry, Chinese Academy of Sciences (CAS), Guangzhou 510640, China; 3 YongZhou Product & Commodity Quality Supervison & Inspection Institute, Yongzhou 425000, China; 13407460777@163.com

**Keywords:** kaolinite, adsorbent, congo red, reconstruct

## Abstract

Organic dyes are widely used in many important areas, but they also bring many issues for water pollution. To address the above issues, a reconstructed kaolinite hybrid compound (γ-AlOOH@A-Kaol) was obtained from raw kaolinite (Kaol) in this work. The product was then characterized by X-ray diffraction (XRD), Fourier-transform infrared (ATR-FTIR), Brunauer-Emmett-Teller (BET), and scanning electron microscopy (SEM), and the absorption properties of γ-AlOOH@A-Kaol for congo red were further studied. The results demonstrated that flower-like γ-AlOOH with nanolamellae were uniformly loaded on the surface of acid-treated Kaol with a porous structure (A-Kaol). In addition, the surface area (36.5 m^2^/g), pore volume (0.146 cm^3^/g), and pore size (13.0 nm) of γ-AlOOH@A-Kaol were different from those of A-Kaol (127.4 m^2^/g, 0.127 cm^3^/g, and 4.28 nm, respectively) and γ-AlOOH (34.1 m^2^/g, 0.315 cm^3^/g, and 21.5 nm, respectively). The unique structure could significantly enhance the sorption capacity for congo red, which could exceed 1000 mg/g. The reasons may be ascribed to the abundant groups of -OH, large specific surface area, and porous structure of γ-AlOOH@A-Kaol. This work provides an efficient route for comprehensive utilization and production of Kaol-based compound materials that could be used in the field of environmental conservation.

## 1. Introduction

Rapid social and economic development has brought great convenience to our daily lives. However, environmental pollution, especially water pollution, is becoming increasingly serious, which has attracted widespread attention in academic and industrial circles [1,2]. Those contaminants, such as organic dyes, are seriously threatening the health and development of humans, plants, and animals [3,4,5]. Therefore, it is necessary to remove those pollutants from wastewater before it is discharged into natural water bodies, which will be beneficial for protecting our environment.

To date, there are some different approaches to address these issues, and adsorption [6], photo-catalytic degradation [3], chelating flocculation [7], chemical precipitation [8], electrochemical oxidation [9], membrane separation [10], and ion exchange [11] have been used to remove these pollutants in wastewater. Among many methods, the adsorption method only requires cheap devices and no harmful byproducts are generated during simple processing to treat wastewater vis-à-vis other methods. Consequently, this method exhibits many advantages, such as a wide applicability, feasible operation, and affordability. 

Among various adsorbents, nano materials (such as carbon nanotubes and graphene oxide) [12,13,14,15], biomass materials (like chitosan and phytic acid) [16,17,18], or waste materials from in our daily lives (like straw and wood) [19,20,21] are the most popular research materials, which depends on their advantages such as efficient removal or degradation and low cost. But nanomaterials and biomass materials are often cost-prohibitive, while the adsorption capacity of waste materials is generally not large. Recently, silicate clays have widely acted as alternative inexpensive adsorbents due to being porous materials with varied sizes and shapes, non-toxic, and they can be easily obtained and regenerated in nature [22,23,24,25].

Acting as one kind of natural clay with low cost, kaolinite (Kaol) has been applied in this field due to its physical and chemical characteristics. It belongs to layer silicate clays with a theoretical formula of Si_2_Al_2_O_5_(OH)_4_ [26,27,28]. The isomorphous substitution in the silica layer of Kaol (with Si^4+^ being replaced by Al^3+^) can form a constant structure with a negative charge, which can act as adsorption sites to remove organic dyes from wastewater solution, relying on the pH value. However, the low adsorption capacity and chemical activity of Kaol limit its further application. The main reasons may be ascribed to the minimal layer spacing (~0.72 nm) of Kaol, strong hydrogen bonding, and extremely low ion exchange capacity vis-à-vis other clay minerals. 

In order to further enhance its adsorption capacity in wastewater containing organic dyes, some work on modifying Kaol has been performed. Especially, Yan et al. [29,30] have deeply explored the adsorption performance of Kaol-based compound materials, which consist of γ-AlOOH and porous silica material. The results indicated that the adsorption capacity for congo red of APTES-modified Kaol-based compound materials or γ-AlOOH nanosheets from Kaol was up to 506 or 786 mg/g, respectively, compared to 6.35 mg/g for raw Kaol. The reason was attributed to the broad range of specific surface areas (S), total pore volumes (V), and positively charged Al-OH groups of Kaol-based compound materials. However, in order to further improve the adsorption ability of Kaol-based compound materials and enhance the high-value utilization of natural clay, the size, structure, and micromorphology of adsorption materials, including γ-AlOOH and porous silica material from Kaol, should be further designed and adjusted. 

In this work, a flower-like γ-AlOOH from Kaol was loaded on the surface of porous silica material to form a hybrid compound (γ-AlOOH@A-Kaol) by hydrothermal synthesis, which possessed a porous structure and Al-OH groups. The physicochemical structure and surface properties of γ-AlOOH@A-Kaol are discussed in detail. The adsorption performance was assessed, including the effects of kind of absorbent, contact time, adsorbent dosage, initial congo red concentration, solution pH, and temperature. Moreover, the mechanism of congo red adsorption was explored and analyzed under the optimal conditions by studying the changes in different factors.

## 2. Results and Discussion

### 2.1. Characterization

**Figure 1:**
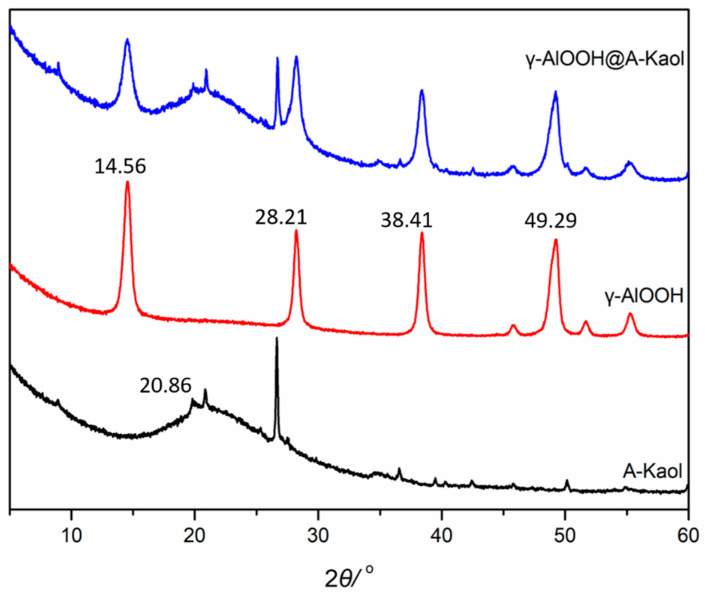
The XRD patterns of A-Kaol, γ-AlOOH, and γ-AlOOH@A-Kaol.

Figure 1 presents the XRD results of A-Kaol, γ-AlOOH, and γ-AlOOH@A-Kaol. A-Kaol showed a peak at 20.86° and a broad band between 15 and 25°, which could be attributed to crystalline and amorphous phases of SiO_2_ [31], respectively. As for γ-AlOOH, the characteristic diffraction peaks were assigned to 14.56, 28.21, 38.41, and 49.29°, which corresponded to the crystal planes of (020), (120), (031), and (200), respectively [32,33,34]. After treatment by γ-AlOOH, it was found that the characteristic diffraction peaks of γ-AlOOH and A-Kaol were also present. However, the peak intensity and width of γ-AlOOH weakened and expanded, respectively. The results indicated that γ-AlOOH likely loaded on the surface of A-Kaol via bonding energy.

**Figure 2:**
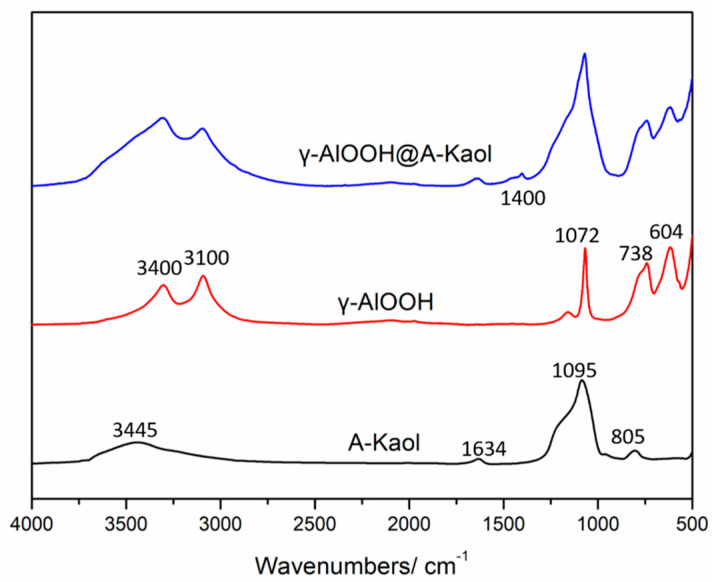
The FTIR spectra of A-Kaol, γ-AlOOH, and γ-AlOOH@A-Kaol.

The FTIR patterns of A-Kaol, γ-AlOOH, and γ-AlOOH@A-Kaol are presented in Figure 2. Two broad peaks at 3445 and 1634 cm^−1^ in A-Kaol showed the physisorption of water on the surface. Peaks at 1095 and 805 cm^−1^ were assigned to Si-O-Si and Si-O stretching [35,36]. The two peaks at 3400 and 3100 cm^−1^ were ascribed to −OH of γ-AlOOH. Meanwhile, the peak at 604 cm^−1^ corresponded to Al-O stretching of γ-AlOOH, and peaks at 738 and 1072 cm^−1^ were attributed to the torsional and symmetric bending modes of Al(OH) [37]. When γ-AlOOH was loaded on the surface of A-Kaol, the characteristic stretching of γ-AlOOH and A-Kaol were also found. However, the intensity and width of the peaks, especially around 3500~3000 and 1200~1000 cm^−1^, were obviously altered. In addition, the ratio of peaks at 3400 and 3100 cm^−1^ was increased, and a new peak at 1400 cm^−1^ emerged along with the above changes. It was indicated that the bond of H-O-Al of γ-AlOOH reacted with the O-Si bond of A-Kaol to form γ-AlOOH@A-Kaol.

**Figure 3:**
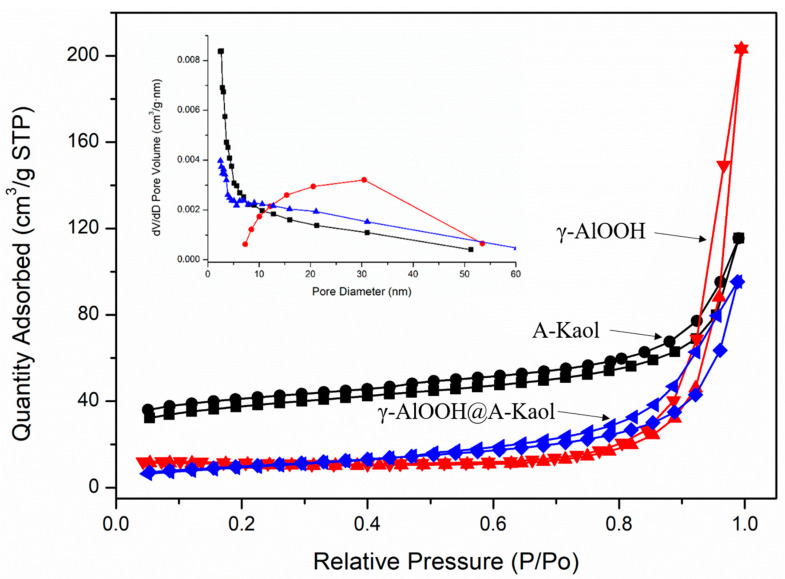
N_2_ adsorption-desorption isotherms of A-Kaol, γ-AlOOH, and γ-AlOOH@A-Kaol.

The N_2_ absorption-desorption isotherms of A-Kaol, γ-AlOOH, and γ-AlOOH@A-Kaol were categorized (Figure 3), and the corresponding key data are presented in Table 1. The results (summarized in Table 1) revealed that the values of S, V, and D of both A-Kaol (127.4 m^2^/g, 0.127 cm^3^/g, and 4.28 nm, respectively) and γ-AlOOH (34.1 m^2^/g, 0.315 cm^3^/g, and 21.5 nm, respectively). After the reaction between γ-AlOOH and A-Kaol, the values of S, V, and D of γ-AlOOH@A-Kaol (36.5 m^2^/g, 0.146 cm^3^/g, and 13.0 nm, respectively) were close to those of γ-AlOOH and A-Kaol, meaning a difference after treatment. All samples exhibited one kind of IV isotherm (with H4 hysteresis loops for A-Kaol and γ-AlOOH@A-Kaol, as well as H3 hysteresis loops for γ-AlOOH), which displayed the existence of both mesopores and macropores. Additionally, A-Kaol and γ-AlOOH@A-Kaol showed a steep slope in the P/P_0_ < 0.01, indicating the existence of micropores. However, the number of micropores for γ-AlOOH was almost zero, judged from the steep slope in the P/P_0_ < 0.01. In addition, the value for γ-AlOOH@A-Kaol was more than three times that for A-Kaol and almost two-thirds smaller than that for γ-AlOOH. The results indicated that the numbers of micropores increased significantly. As for the BJH D distribution curves, A-Kaol and γ-AlOOH had mesopores centered at 1~10 and 12~40 nm, respectively. While for γ-AlOOH@A-Kaol, the shape of the curve and numbers of little mesopores (2~10 nm) and mid-sized mesopores (10~25 nm) were obviously different compared to γ-AlOOH and A-Kaol, which corresponded to the variation in S and V values through the assembly of γ-AlOOH and A-Kaol. Even the S value of A-Kaol was the largest compared to the other two samples, and the D value was the smallest. This structure was not conducive to the adsorption of congo red. As for γ-AlOOH, although its S value was similar to that of γ-AlOOH@A-Kaol, the values of D and V were too large, which may not be conducive to the adsorption of congo red. 

**Figure 4:**
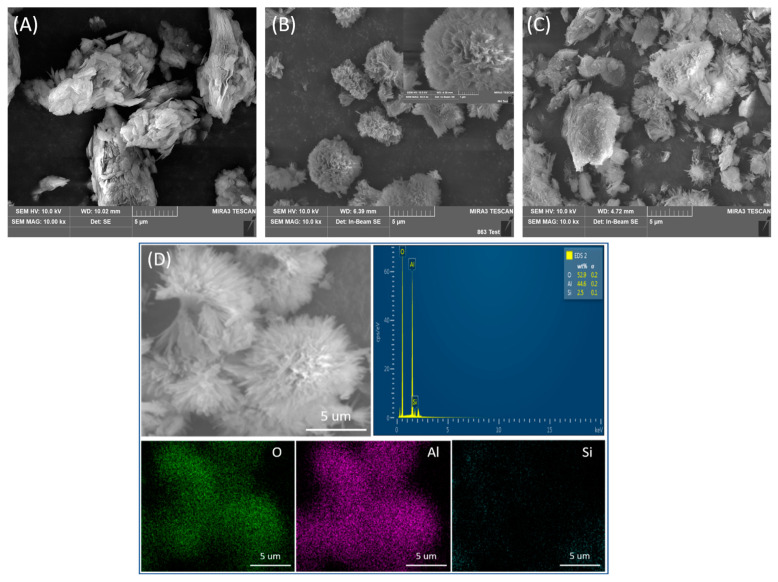
SEM photos of A-Kaol (**A**), γ-AlOOH (**B**), and γ-AlOOH@A-Kaol (**C**), and the EDS and mapping results of γ-AlOOH@A-Kaol (**D**).

The micromorphology of A-Kaol, γ-AlOOH, and γ-AlOOH@A-Kaol was observed under SEM, and the results are shown in Figure 4. It can be clearly seen that the layered structure of A-Kaol (Figure 4A), flower-like γ-AlOOH (see the inset of Figure 4B), and further the flower-like γ-AlOOH were uniformly grown on the surface A-Kaol to form γ-AlOOH@A-Kaol (Figure 4C). The corresponding element contents of O, Al, and Si in γ-AlOOH@A-Kaol were 52.9, 44.6, and 2.5%, respectively (Figure 4D), and the mapping of the Al, O, and Si elements demonstrated that the elements were distributed homogeneously on γ-AlOOH@A-Kaol. The above results illustrated the successful introduction of γ-AlOOH on the surface of A-Kaol.

### 2.2. Adsorption Properties of γ-AlOOH@A-Kaol

**Figure 5:**
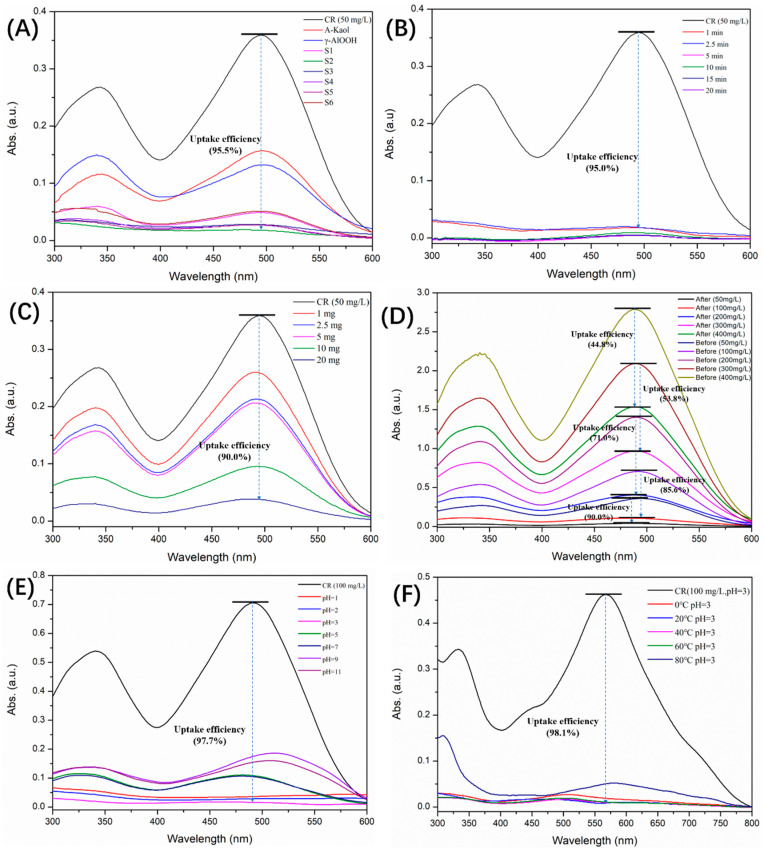
Adsorption properties of γ-AlOOH@A-Kaol under different conditions: (**A**) Samples, (**B**) Time, (**C**) Quality, (**D**) Concentration, (**E**) pH, (**F**) Temperature.

In this work, the congo red adsorption tests (20 mL, 50 mg/L, pH = 7, and 20 °C) were first performed using 25 mg γ-AlOOH@A-Kaol samples with different mass ratios under 25 min (Figure 5A). It was shown that sample S2 (mass ratio of γ-AlOOH and A-Kaol was 0.2) had the highest uptake efficiency (95.5%) vis-à-vis other samples and raw materials. So, sample S2 was chosen for the next research work. 

Figure 5B shows the effect of adsorption time on congo red with the other conditions unchanged (20 mL, 50 mg/L, pH = 7, and 20 °C). It demonstrates that γ-AlOOH@A-Kaol (25 mg) had excellent uptake efficiency. Even in a very short time (like 1 min), the uptake efficiency still could reach 95.0%. Therefore, the adsorption time was set as 1 min in the following experiments. Therefore, the selection of optimum adsorbent quality (γ-AlOOH@A-Kaol, 20 mg) was based on the optimal uptake efficiency (Figure 5C) under these conditions (20 mL, 50 mg/L, pH = 7, 20 °C, and 1 min). In other words, the uptake efficiency reached 90.0% under the above conditions. Thus, the UV pattern of γ-AlOOH@A-Kaol in the adsorption concentration control group (20 mL, pH = 7, 20 °C, 20 mg, and 1 min) was then measured, and the results are shown in Figure 5D. It demonstrates that 100 mg/L congo red could be efficiently reduced to 11.6 mg/L (uptake efficiency nearly about 90.0%), while the uptake efficiency of the samples with high concentrations (such as 200, 300, and 400 mg/L) dropped to below 71%. In addition, the uptake efficiency of the low concentration sample was just 90%. So, 100 mg/L congo red was selected as the next research object. The influence of factors such as pH (Figure 5E) and adsorption temperature (Figure 5F) were then carefully investigated based on the above optimum conditions, indicating that the best adsorption effect was achieved at pH = 3 and 20 °C, respectively. 

**Figure 6:**
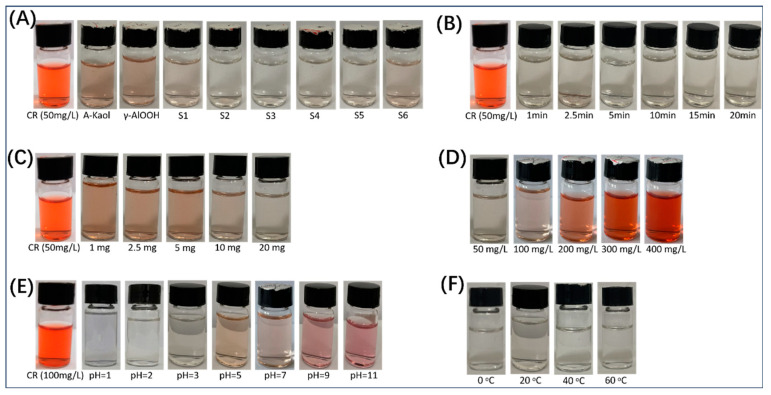
The digital photographs of γ-AlOOH@A-Kaol after adsorption: (**A**) Samples, (**B**) Time, (**C**) Quality, (**D**) Concentration, (**E**) pH, (**F**) Temperature.

In addition, the corresponding digital photographs (Figure 6A–E) also illustrated the adsorption effect of γ-AlOOH@A-Kaol for congo red under different conditions. Therefore, these phenomena demonstrated that 20 mg γ-AlOOH@A-Kaol (S2) showed the highest uptake efficiency under these conditions (adsorption time = 1 min, initial concentration = 100 mg/L, solution pH = 3, and solution temperature = 20 °C), which could reach 98.1% compared to the original concentration of congo red (100 mg/L).

### 2.3. Adsorption Isotherm and Kinetic Models

**Figure 7:**
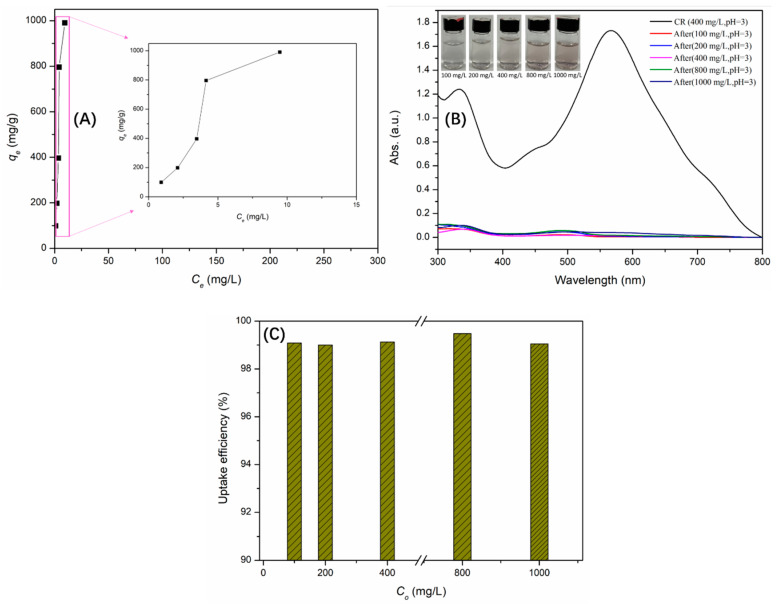
The sorption isotherms (**A**), sorption capacity (**B**) and uptake efficiency (**C**) of γ-AlOOH@A-Kaol.

According to the above results, γ-AlOOH@A-Kaol exhibited a significant potential for adsorption of congo red. Figure 7A shows the results of the sorption isotherms at pH = 3 (20 °C), which revealed that γ-AlOOH@A-Kaol held a larger sorption capacity (more than 1000 mg/g) based on its adsorption tests (Figure 7B). In addition, the uptake efficiency of γ-AlOOH@A-Kaol exceeded 99%, ranging from 100 to 1000 mg/L of the initial congo red concentration, as shown in Figure 7C, suggesting that γ-AlOOH@A-Kaol could efficiently be applied for broad concentrations of congo red under the above conditions. 

**Figure 8:**
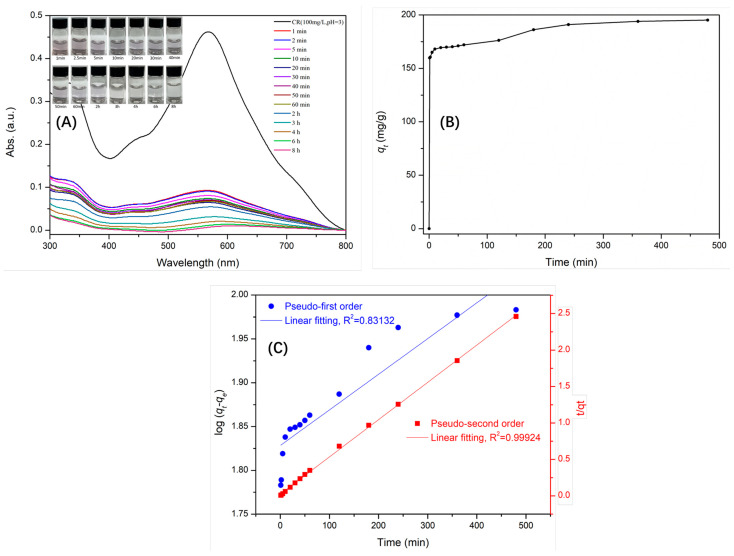
The kinetics uptake efficiency (**A**), adsorption capacity (**B**), and adsorption kinetics (**C**) of γ-AlOOH@A-Kaol.

The contents of congo red in solution were recorded through kinetic experiments at various times. The results revealed that the uptake efficiency and adsorption capacity of γ-AlOOH@A-Kaol reached 80.0% (Figure 8A) and 159.8 mg/g (Figure 8B) within 1 min, respectively, and then achieved sorption equilibrium within the next 360 min (Figure 8B). This phenomenon showed that the flower-like nanostructure compound of γ-AlOOH@A-Kaol could favor the adsorption of congo red. Meanwhile, the corresponding digital photographs (Figure 8A) clearly illustrated the adsorption effect of γ-AlOOH@A-Kaol for congo red.

The adsorption kinetics were investigated to evaluate the mechanism and rate of congo red molecule transfer onto the γ-AlOOH@A-Kaol compound surface from the liquid solution, and the results are shown in Figure 8C and Table 2. Figure 8C presents the data and fitting models of pseudo-first-order (PFO) and pseudo-second-order (PSO) kinetics. As shown in Table 2, the values of R^2^ for PFO and PSO were 0.83132 and 0.99924, respectively. It was found that the PSO kinetic model better fit the data than the PFO kinetic model, meaning that the PSO kinetic model could well illustrate the adsorption of congo red onto the γ-AlOOH@A-Kaol surface. Therefore, the adsorption rate constant of γ-AlOOH@A-Kaol was calculated to be 0.0013 g/(mg·min) from the PSO kinetic model.

### 2.4. Adsorption Mechanism

**Figure 9:**
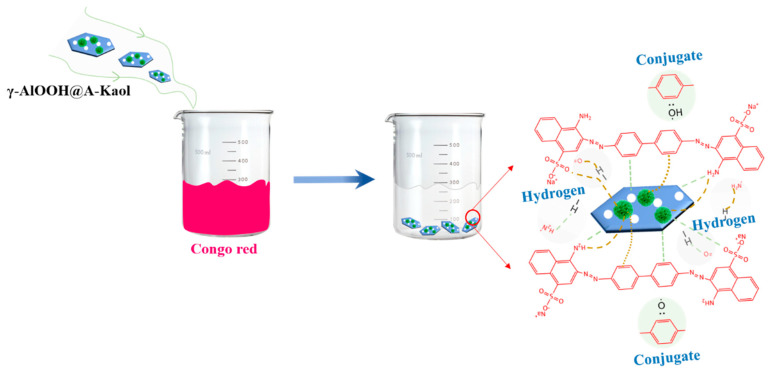
Possible adsorption mechanism of γ-AlOOH@A-Kaol in congo red solution.

Based on the structure of γ-AlOOH@A-Kaol and the above results, the adsorption mechanism of γ-AlOOH@A-Kaol for congo red is shown in Figure 9, which includes hydrogen bonding and conjugate adsorption, as well as the schematic diagram.

## 3. Experimental

### 3.1. Materials

Kaol was provided by Xing Yi Mineral Processing Plant (Shijiazhuang, China); sulfuric acid was supplied by Beijing Chemical Factory (Beijing, China); and urea and anhydrous ethanol were purchased from Aladdin Reagent Co., Ltd. (Shanghai, China). Congo red was obtained from Sinopharm Chemical Reagent Co., Ltd. (Shanghai, China).

### 3.2. Preparation of γ-AlOOH@A-Kaol

Kaol was calcined at 850 °C for 2 h to obtain metakaolinite. Metakaolinite was then treated at 90 °C with 4 mol/L sulfuric acid solution for 1 h to separate the silica source and aluminum source, followed by filtration. The silica was washed with distilled water before being dried at 80 °C for 24 h to prepare a solid filter cake. Then, it was calcined at 400 °C for 2 h to obtain the silica material (A-Kaol) from Kaol. The aluminum source was placed in an ice bath after spinning to obtain white crystals (crude product of Al_2_(SO_4_)_3_∙18H_2_O), which were then was washed with anhydrous ethanol before being dried at 80 °C for 24 h to further purify the white crystals. 

**Figure 10:**
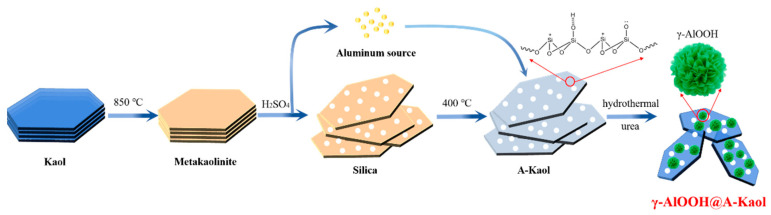
The synthetic process of γ-AlOOH@A-Kaol.

Next, 3.0 g Al_2_(SO_4_)_3_∙18H_2_O and 1.08 g urea were added into the hydrothermal reactor, and then a certain amount of A-Kaol was introduced after uniform stirring, followed by transformation in a muffle furnace for 8 h at 240 °C. Finally, it washed with distilled water before being dried for 24 h at 80 °C to prepare the target materials. The detailed mass ratios and synthetic process are shown in Table 3 and Figure 10, respectively.

### 3.3. Congo Red Adsorption Experiments

A certain mass of γ-AlOOH@A-Kaol was added to the solution containing congo red, and the mixture was shaken under various predetermined conditions under static conditions. First, the samples with different mass ratios and raw materials were explored in the congo red adsorption tests, and the adsorption performance of γ-AlOOH@A-Kaol was further explored in congo red solution. The adsorption tests were then carried out at different adsorption times, adsorbent dosages, initial concentrations, solution pH, and temperature based on the results of the previous test for each experiment. The detailed adsorption tests are shown in Section 2.2. *Adsorption properties of γ-AlOOH@A-Kaol*.

The congo red solution was then centrifuged, and the supernatant liquid was tested by UV-Vis spectrophotometry (Shimadzu, UV-1800, Kyoto, Japan). Each adsorption experiment was carried out three times, and the final data were obtained from the average values with errors within 3%. The adsorption capacity of congo red on γ-AlOOH@A-Kaol and its raw materials was estimated according to the following formulas:

(1) $$q_{t}=\frac{\left(C_{o}-C_{t}\right)}{m}\;V$$

(2) $$q_{e}=\frac{\left(C_{o}-C_{e}\right)}{m}\;V$$

where *q_e_* (mg/g) and *q_t_* (mg/g) are the equilibrium adsorption capacity and the adsorption capacity at time *t* (min), respectively. The initial concentration, equilibrium concentration, and concentration at time *t* are named as *C_o_* (mg/L), *C_e_* (mg/L), and *C_t_* (mg/L), respectively. In addition, *V* (L) and *m* (g) are the volume of the solution and mass of the adsorbent, respectively.

Moreover, the corresponding experiments for the adsorption kinetic tests were performed based on the experimental data using the following equations:

(3) $$\mathrm{Pseudo}-\mathrm{first}-\mathrm{order}:\;q_{t}=q_{e}(1-e^{-K1\cdot t})$$

(4) $$\mathrm{Pseudo}-\mathrm{second}-\mathrm{order}:\;=\frac{t\cdot K2\cdot q_{e\;}^{2}}{1+t\cdot K2\cdot q_{e}}$$

where *K*1 (min^−1^) and *K*2 (g/(mg∙min)) are the rate constants for pseudo-first-order and pseudo-second-order reactions, respectively.

### 3.4. Standard Curve

**Figure 11:**
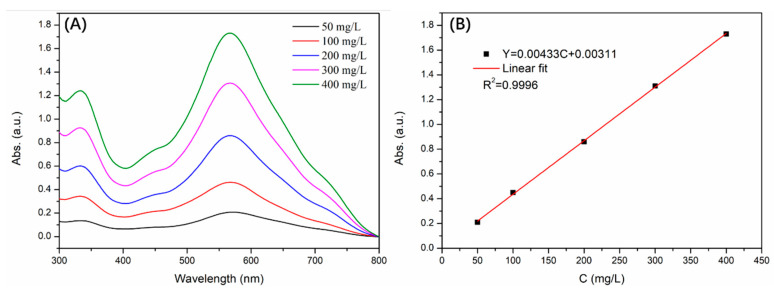
The absorbance tests of congo red standard solution with gradient concentrations (**A**) and the corresponding fitting curve (**B**).

In this section, the absorbance experiments were conducted under the optimal adsorption conditions based on the above results. Therefore, the absorbance of 100 mg/L congo red standard solution was first tested using a UV-Vis spectrophotometer to determine the wavelength at the maximum absorbance value. Thus, the subsequent experiments were conducted at this wavelength. Subsequently, the absorbance values of congo red standard solution with gradient concentrations were measured, and the corresponding results are exhibited in Figure 11. It was shown that the relevant index R^2^ was 0.9996 and the fitting curve was y = 0.00433C + 0.00311.

### 3.5. Characterization of γ-AlOOH@A-Kaol 

The X-ray diffraction (XRD) spectra were recorded with a D/Max-2500 diffractometer (Rigaku, Tokyo, Japan). The Cu Kα radiation source was employed using a generator voltage of 40 KV and a current of 20 mA (*λ* = 0.154 nm). Samples were measured from 5 to 60° at a scan rate of 2°/min. 

The Fourier-transform infrared (ATR-FTIR) spectra were recorded using a Nicolet IS 10 FTIR (Thermo Nicolet, Waltham, MA, USA) spectrometer at a resolution of 1 cm^−1^ for 32 times.

The N_2_ adsorption-desorption isotherms were determined at 77 K using a Micromeritics ASAP 2060 (Micromeritics, Norcross, GA, USA). Samples were degassed at 100 °C for at least 6 h prior to Brunauer-Emmett-Teller (BET) measurements. The S, V, and average pore size (D) of the raw materials and γ-AlOOH@A-Kaol were then estimated.

The morphology of γ-AlOOH@A-Kaol and its raw materials was observed by a MIRA3 (Tescan, Brno, Czech Republic) scanning electron microscope (SEM) under a voltage of 10 kV. The relative element compositions of A-Kaol, γ-AlOOH, and γ-AlOOH@A-Kaol were synchronously detected by energy dispersive spectroscopy (EDS).

## 4. Conclusions

In this work, a Kaol-based hybrid compound (γ-AlOOH@A-Kaol) was successfully prepared via γ-AlOOH loaded on the surface of A-Kaol, and then it was introduced into wastewater solution containing congo red to remove pollutants. The results showed that γ-AlOOH@A-Kaol had high adsorption capacity for congo red under specific conditions (adsorption time = 1 min, initial concentration = 100 mg/L, solution pH = 3, and solution temperature = 20 °C) and fast sorption kinetics. Meanwhile, the adsorption mechanism was also discussed. This work can provide a method to obtain an adsorbent for congo red from Kaol. Therefore, it could serve as a favorable candidate in environmental remediation fields.

## Figures and Tables

**Table 1:** BET results of A-Kaol, γ-AlOOH and γ-AlOOH@A-Kaol.

Samples	S (m^2^g^−1^) ^a^	V (cm^3^g^−1^) ^b^	D (nm) ^c^
A-Kaol	127.4	0.127	4.28
γ-AlOOH	34.1	0.315	21.5
γ-AlOOH@A-Kaol	36.5	0.146	13.0

^a^ Calculated with the BET method; ^b^ Obtained from BJH desorption; ^c^ Obtained from desorption by BET

**Table 2:** The key data of adsorption kinetics for γ-AlOOH@A-Kaol.

PFO			PSO		
q_e cal_ (mg/g)	K_1_	R^2^	q_e cal_ (mg/g)	K_2_	R^2^
67.3	9.36 × 10^−4^	0.83132	195.3	0.0013	0.99934

**Table 3:** Formulations of raw materials for γ-AlOOH@A-Kaol.

Samples	m/Al_2_(SO_4_)_3_·18H_2_O	m/Urea	MM *	m/A-Kaol
A-Kaol	0	0	0	0
γ-AlOOH	3 g	1.08 g	0	0
S1	3 g	1.08 g	1/5	0.816 g
S2	3 g	1.08 g	1/10	0.408 g
S3	3 g	1.08 g	1/20	0.204 g
S4	3 g	1.08 g	1/40	0.102 g
S5	3 g	1.08 g	1/80	0.051 g
S6	3 g	1.08 g	1/160	0.0255 g

* Mass ratio of A-Kaol relative to total mass of Al_2_(SO_4_)_3_·18H_2_O and urea.

## Data Availability

The data are available upon request.
